# Structural Studies of Water-Insoluble β-Glucan from Oat Bran and Its Effect on Improving Lipid Metabolism in Mice Fed High-Fat Diet

**DOI:** 10.3390/nu13093254

**Published:** 2021-09-18

**Authors:** Shoujuan Yu, Jun Wang, Yixuan Li, Xifan Wang, Fazheng Ren, Xiaoyu Wang

**Affiliations:** 1 Key Laboratory of Functional Dairy, Ministry of Education, College of Food Science and Nutritional Engineering, China Agricultural University, Beijing 100083, China; yshoujuan@163.com (S.Y.); wangjun1@cau.edu.cn (J.W.); 2 Key Laboratory of Precision Nutrition and Food Quality, Department of Nutrition and Health, China Agricultural University, Beijing 100083, China; liyixuan9735@126.com (Y.L.); renfazheng@263.net (F.R.); 3 Department of Obstetrics and Gynecology, Columbia University, New York, NY 10032, USA; wangxfan@126.com

**Keywords:** water-insoluble β-glucan, structure characterization, physicochemical properties, lipid metabolism

## Abstract

Water-insoluble β-glucan has been reported to have beneficial effects on human health. However, no studies have thoroughly characterized the structure and function of water-insoluble β-glucan in oat bran. Thus, the structure and effect of water-insoluble β-glucan on weight gain and lipid metabolism in high-fat diet (HFD)-fed mice were analyzed. First, water-insoluble β-glucan was isolated and purified from oat bran. Compared with water-soluble β-glucan, water-insoluble β-glucan had higher DP3:DP4 molar ratio (2.12 and 1.67, respectively) and molecular weight (123,800 and 119,200 g/mol, respectively). Notably, water-insoluble β-glucan exhibited more fibrous sheet-like structure and greater swelling power than water-soluble β-glucan. Animal experiments have shown that oral administration of water-insoluble β-glucan tended to lower the final body weight of obese mice after 10 weeks treatment. In addition, water-insoluble β-glucan administration significantly improved the serum lipid profile (triglyceride, total cholesterol, high-density lipoprotein cholesterol, and low-density lipoprotein cholesterol levels) and epididymal adipocytes size. What is more, water-insoluble β-glucan reduced the accumulation and accelerated the decomposition of lipid in liver. In conclusion, water-insoluble β-glucan (oat bran) could alleviate obesity in HFD-fed mice by improving blood lipid level and accelerating the decomposition of lipid.

## 1. Introduction

Mixed-linkage β-glucan is a high-molecular-weight polymer composed of monomeric β-D-glucopyranose via β-1,3- and β-1,4-glycosidic bonds [1]. β-Glucan is mainly found in the aleurone layer, sub-dextrin layer, and cell wall of some cereal endosperms, such as oats, barley, and rice. Oat bran is the part of oat obtained after removal of the endosperm; thus, oat bran is expected to be the best source of β-glucan. The extraction of β-glucan from oats was carried out by hot water, alkali liquor, combined with enzymatic or ultrasonic physical methods. For the purification of β-glucan, ammonium sulfate or ethanol reagents were generally used and followed by the gel chromatography method [2]. β-Glucan can be divided into water-soluble and water-insoluble fractions according to its solubility in water. Most studies have focused on water-soluble β-glucan, while few have focused on water-insoluble β-glucan.

Oat β-glucan has been shown to have several beneficial effects on human health [3,4]. For example, many studies have shown that water-soluble β-glucan has various physiological functions, including lowering plasma cholesterol and postprandial blood glucose and improving intestinal microecology [5,6,7]. In recent years, water-insoluble β-glucan has been shown to have some physiological functions. Shen et al. [4] found that water-insoluble β-glucan could prevent colon cancer in a dose-dependent manner. Dong et al. [3] found that water-insoluble β-glucan could reduce obesity, which is more effective in mediating weight loss than water-soluble β-glucan, suggesting that water-insoluble β-glucan may have potential applications in weight management. However, there was limited information about the structure of water-insoluble β-glucan in oat bran, which restricts the study of its physiological function.

The physiological function of β-glucan is related to its physicochemical characteristics, which depend on its structure [8]. Studies have shown that the molecular structures of β-glucan from different grains are similar; however, variations in molecular weight, the β-1,4- to β-1,3- bond ratio, and block structures (ratio of cellotriosyl/cellotetraosyl units) have been observed [9,10]. These structural differences may explain the diverse physicochemical characteristics of these molecules. For example, β-glucan samples with higher molar mass exhibited higher viscosity [11]. Moreover, cellotriosyl units and longer β-1,4-bond segments contribute to grain β-glucan viscosity and gel formation [12]. The physicochemical characteristics of β-glucan are particularly important for the study of its physiological functions. Many studies have demonstrated a relationship between the viscosity of oat-soluble β-glucan and glycemic response [13,14,15]. However, the structure, physicochemical characteristics, and functions of water-insoluble β-glucan in oat bran have not been elucidated.

Considering the previously known effects of water-insoluble β-glucan on human health, it is necessary to systematically elucidate its structure and function in obese mice. Therefore, in this study, water-insoluble β-glucan was extracted and purified from oat bran, and its structure, physicochemical characteristics, and function of preventing weight gain and improving lipid metabolism were studied.

## 2. Materials and Methods

### 2.1. Materials

Oat bran was purchased from Tong Yuan Gong He Co., Ltd. (Yantai, China). TermamylSC and amyloglucosidase were obtained from Novozymes Co., Ltd. (Bagsvaerd, Denmark). Pepsin and trypsin were obtained from Amresco Co., Ltd. (Solon, OH, USA). Lichenase was purchased from Megazyme (Wicklow, Ireland). Mannose, glucose, galactose, xylose, arabinose, and fucose standard were obtained from Sigma-Aldrich Chemical Co. (St. Louis, MO, USA). DEAE52 and SephadexG200 were purchased from GE Healthcare. All other chemicals used were of analytical grade.

### 2.2. Separation of Water-Insoluble Dietary Fiber from Oat Bran

Insoluble dietary fiber was prepared according to the previously reported methods with slight modifications [16]. The extraction process is shown in Figure S1a. The specific extraction method is shown in Supplementary Material Section S2.2. The precipitate was oat-insoluble dietary fiber. The supernatant was precipitated by 70% ethanol, and it was defined as water-soluble β-glucan concentration.

### 2.3. Extraction and Purification of Oat Water-Insoluble β-Glucan

Water-insoluble β-glucan was isolated using the previously reported method [2,17]. The entire process is shown in Figure S1b. The specific extraction method is shown in Supplementary Material Section S2.3. After insoluble dietary fiber was extracted with barium hydroxide, the insoluble material was subjected to additional extraction with potassium hydroxide (Fraction 1). Then, the supernatant was separated and precipitated with 70% ethanol to obtain water-insoluble β-glucan mixture (Fraction 2). Then, DEAE-52 cellulose and SephadexG200 were used for the purification of water-soluble and -insoluble β-glucan [18,19,20]. Finally, the purified sample was freeze-dried in vacuum.

### 2.4. Structural Identification and Characterization of Water-Insoluble β-Glucan

#### 2.4.1. Monosaccharide Composition and Molecular Weight of Water-Insoluble β-Glucan

Monosaccharides were quantified after acid hydrolysis by high-performance liquid chromatography, as previously described [21]. Samples and monosaccharide standards were hydrolyzed with trifluoroacetic acid and subjected to chromatography on an Ultimate 3000 instrument with a Xtimate C18 column (4.6 mm × 200 mm × 5 μm). The following settings were used: column temperature, 30 °C; flow rate, 1 mL/min; detection wavelength, 250 nm; injection volume, 20 μL; mobile phase A, 0.05 M potassium dihydrogen phosphate solution (pH 6.70 with sodium hydroxide solution); mobile phase B, acetonitrile; gradient, 83% A/17% B.

Gel permeation chromatography was performed on Shimadzu-20A Gel permeation chromatograph equipped with Shimadzu RID-20A refractive index detector (Shimadzu, Japan) and LC20 high-performance liquid chromatography pump. The gel chromatography column was TSKgel GMPWXL (Tosoh; Tokyo, Japan), and the GPC chromatography workstation was HW-2000. A 0.1 N NaNO_3_ (0.06% sodium azide) solution was used as the mobile phase and eluted at a flow rate of 0.6 mL/min. The temperatures of the column and detector were both maintained at 35 °C during the determination process [22]. Narrow-distribution polyethylene glycol (molecular weight: 580,000 Da, 146,000 Da, 44,200 Da, 1,000 Da, and 600 Da, respectively) was used as standard. The molecular weight of β-glucan was obtained after universal calibration according to the standard curve and Flory’s characteristic viscosity number theory.

#### 2.4.2. Oligosaccharide Content (DP3:DP4)

The purified β-glucan samples were prepared with 2.15 mL (0.5% *w*/*v*) of ultrapure water. The suspension was incubated in boiling water with continuous stirring until the β-glucan was fully solubilized. After cooling to room temperature, the pH was adjusted to 6.5 with sodium hydroxide, and 1.35 mL lichenase (150 U) was added. Then, the mixture was incubated at 50 °C for 1 h and stirred every 20 min. The hydrolysate was heated in boiling water bath to inactivate the enzyme and centrifuged (Thermo Scientific™ Sorvall™ LYNX 6000, Thermo; Waltham, MA, USA) at 4580× *g* for 20 min [23,24]. Measurements were performed with MALDI-TOF MS (Bruker; Karlsruhe, Germany) equipped with a pulsed N_2_ laser (337 nm). An accelerating voltage of 20 kV was used, and DHB was used as the matrix. Bruker’s mass spectrometry calibration solution was used to calibrate the mass spectrometer.

#### 2.4.3. Nuclear Magnetic Resonance (NMR) Spectroscopy Measurement

For NMR analysis, 30 mg of the dried polysaccharide was dissolved in 1 mL of D_2_O, completely dissolved, and lyophilized; this procedure was repeated twice, and the final sample was dissolved in 0.5 mL of D_2_O. ^1^H NMR and ^13^C NMR were obtained using Bruker NMR spectrometer (Bruker; Karlsruhe, Germany), operating at 600 MHz for protons. The measurements were performed at 80 °C [25]. The scan time was 128 (2048), and the delay between pulses was 4 s for ^1^H NMR (^13^C NMR). The external standard was tetramethylsilane (TMS).

#### 2.4.4. Fourier-Transform Infrared (FT-IR) Spectroscopy

The structures of water-soluble and -insoluble β-glucan were confirmed by FT-IR spectroscopy (Spectrum 100; PerkinElmer; Waltham, MA, USA) at a resolution of 4 cm^−1^ in the range 400–4000 cm^−1^ and referenced against air. The samples were dried in an oven at 105 °C for 2 h and then placed in a dryer for 1 h. Samples (2 mg) and KBr (150 mg) were mixed and ground and then pressed into pellets under a pressure of 20–30 MPa.

#### 2.4.5. X-ray Diffraction

The freeze-dried sample was ground into a powder with a particle size of less than 70 μm (pass 200 mesh). An X-ray diffractometer (D8 Advance; Bruker; Karlsruhe, Germany) was used to analyze the crystalline structures of β-glucan samples at an operating voltage of 40 kV and an incident current of 40 mA [26]. The Cu target wavelength was 1.5406 angstroms. The angular region was scanned from 5° to 70° with a step width of 0.01° and a speed of 6°/min.

#### 2.4.6. Scanning Electron Microscopy (SEM)

The microstructure of the samples was examined by SEM (SU8020; Hitachi; Tokyo, Japan). Samples were freeze-dried from ultrapure water. Then, the samples were sputter-coated with Au Pd for 60 s at a voltage of 10 kV prior to obtaining an image [25].

### 2.5. Physicochemical Characteristics of Water-Insoluble β-Glucan

#### 2.5.1. Swelling Power

Swelling power was determined according to the method of Sun et al. [22]. Briefly, 0.2 g of β-glucan was dissolved in 10 mL of ultrapure water and incubated in a water bath at 70 °C for 10 min with continuous stirring at 500 r/min. Then, samples were transferred to a boiling water bath for 10 min. Samples were cooled to room temperature and centrifuged at 1700× *g* for 4 min. Swelling power was expressed as the ratio of wet sediment weight to dry sample weight.

#### 2.5.2. Fat-Binding Capacity

For analysis of fat-binding capacity, 0.2 g (dry weight) β-glucan was added to 10 mL soy oil and mixed using an Ultraturrax homogenizer (T25; IKA; Staufen, Germany) at 10,000 rpm for 1 min. Then, samples were stored at room temperature for 1 h, with stirring every 15 min, and centrifuged at 1600× *g* for 20 min. Then, the precipitate was weighed (wet weight). Fat-binding capacity = wet weight/dry weight [27,28].

#### 2.5.3. Gel Texture

Gel texture was determined according to the method of De Souza et al. [28] with minor modifications. The gel concentration was 12%, which was prepared in ultrapure water with incubation in a water bath at 90 °C for 30 min. Then, samples were cooled in ice water and placed at 4 °C for 24 h. The gel texture was determined using a texture analyzer (TMS-Pro; FTC; Sterling, VA, USA). The gels were compressed to 50% of their height using a cylindrical probe (10 mm in diameter [P/10]) at a speed of 1 mm·s^−1^, at 25 °C.

#### 2.5.4. Determination of Rheological Properties

The samples were prepared as described by De Moura et al. [27]. β-glucan was prepared in a 3% (*w*/*v*) solution by dissolving the samples in ultrapure water with constant stirring at 80 °C for 1 h. Then, samples were placed in a 4 °C refrigerator for 12 h. Apparent viscosity was measured at 25 °C with a rheometer (TA 1500; TA; New Castle, DE, USA) by varying the shear rate from 0.01 to 1000 s^−1^.

### 2.6. Effect of Water-Insoluble β-Glucan on Weight Gain and Lipid Metabolism in HFD-Fed Mice

#### 2.6.1. Animal Experiments

**Figure 1:**
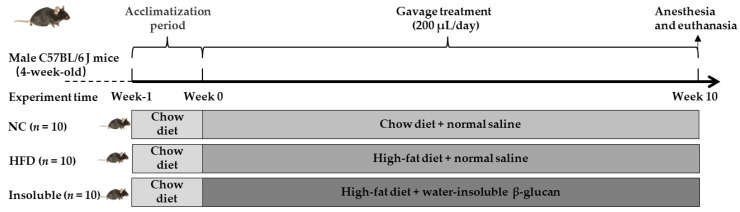
Overview of experimental design.

All animal studies were approved by the Animal Experimentation Ethics Committee of the Pony Testing International Group Co., Ltd. (Beijing, China) on 10 November 2020. The ethic approval code for animal studies were PONY-2020-FL-75. A total of 30 male C57BL/6 J mice (4 weeks old) were provided by Beijing HFK Bioscience Co. Ltd (Beijing, China). The experiment was carried out at Pony Testing International Group Co., Ltd. (Beijing, China). All mice were maintained on a standard diet for 1 week and housed under a standard animal housing facility with an air-conditioned room (22–25 °C), 60% relative humidity, and an artificial 12 light/12 dark cycle (a schematic of the experimental design was provided in Figure 1). The mice were randomly divided into three groups (n = 10): mice fed a chow diet (cat. no. D12450B) were considered as the normal control group (NC group, 10% energy from fat); mice fed a high-fat diet (cat. no. D12451) were considered as the obesity model group (HFD group, 45% energy from fat); mice fed a high-fat diet and given oral gavage with 200 μL water-insoluble β-glucan (1 g·kg^−1^·BW) daily were considered as Insoluble group. These diets were obtained from Research Diets Co., Ltd. (New Brunswick, NJ, USA), and the composition of these diets was shown in Supplementary materials (Table S1). Meanwhile, the NC and HFD groups were given oral administration of normal saline. After 10 weeks of intervention, mice were anesthetized by CO_2_ exposure and killed by cervical dislocation.

#### 2.6.2. Determination of Body Weight and Body Fat Content

The body weight and food intake were measured weekly by a calibrated weighing scale. The body fat and lean tissue to body weight of mice were determined by the nuclear magnetic resonance system using Body Composition Analyzer MiniQMR23-060H-I (Shanghai Niumag Corporation; Shanghai, China).

#### 2.6.3. Serum and Tissue Lipid Profile Analysis

Blood collected from the angular vein was placed at room temperature for 2 h and then centrifuged at 3000× *g* for 15 min at 4 °C to obtain serum. Total cholesterol, triglycerides, low-density lipoprotein cholesterol (LDL-C), and high-density lipoprotein cholesterol (HDL-C) in serum were determined by the total cholesterol assay kit (cat. no. 14162008), triglycerides assay kit (cat. no. 14172006), LDL-C assay kit (cat. no. 14202005), HDL-C assay kit (cat. no. 10500463), and Chemistry Analyzer (BS-350E) provided by Shenzhen Mindray Biomedical Electronics Co., Ltd. (Shenzhen, China).

The epididymal adipose tissues were washed with phosphate-buffered saline and fixed in 10% formalin solution. Tissues were embedded in paraffin and cut into tissue slices with thickness of 5 μm. Finally, the tissues were stained with hematoxylin and eosin (H&E) and photographed under an optical microscope (Leica; Wetzlar, Germany). Image J software was used to obtain the area of each adipocyte in the field of view, and the size of one adipocyte was calculated by dividing the total cell area by the number of cells.

#### 2.6.4. Western Blot Analysis

The protein expression levels of fatty acid synthase (FAS) and hormone-sensitive lipase (HSL) in liver tissues were analyzed by western blot. Fatty acid synthase antibody (cat. no. 3189S) and hormone-sensitive lipase antibody (cat. no. 4107S) were obtained from Cell Signaling Technology Co., Ltd. (Boston, MA, USA). Beta actin polyclonal antibody (cat. no. 20536-1-AP) was purchased from Proteintech Co., Ltd. (Wuhan, China). Total proteins from tissues were isolated using RIPA lysis buffer with 1% phenylmethane sulfonyl fluoride (Beyotime; Shanghai, China) and measured using the BCA Protein Assay Kit (Thermo; Waltham, MA, USA). Protein at the same concentration was separated by SDS-PAGE and then transferred to polyvinylidene fluoride (PVDF) membranes (Millipore; Boston, MA, USA). The PVDF membranes were sequentially incubated in primary and secondary antibodies. The development of the PVDF membranes was conducted in accordance with the instructions for the ECL Plus Ultrasensitive Luminescent Liquid (Solarbio; Beijing, China), and protein bands were obtained using Amersham AI600 Chemiluminescent Imaging System (GE Healthcare; Chicago, IL, USA).

### 2.7. Statistical Analysis

Statistical analysis was performed using Origin version 9.1 (Origin Lab Institute, Inc., Cary, NC, USA) and SPSS 20 software (SPSS Inc., Chicago, IL, USA). For structural experiments, paired-samples t-test was used to test for differences between the means. For animal experiments, data were analyzed by one-way ANOVA. Different letters above bars indicate significant differences (*p* < 0.05; Tukey’s post-ANOVA test).

## 3. Results and Discussion

### 3.1. Monosaccharide Composition and Molecular Weight of Water-Insoluble β-Glucan

Monosaccharide composition was measured by HPLC. Water-insoluble β-glucan comprised glucose, xylose, and arabinose in the ratio of 91.60 ± 0.44%, 2.02 ± 0.44%, and 1.86 ± 0.47%, which was similar to water-soluble β-glucan (91.89 ± 0.43%, 2.13 ± 0.33%, 1.81 ± 0.25%). This indicated that the β-glucan extracted and purified from oat bran was mainly composed of glucose. The water-insoluble β-glucan in our study had higher glucose content than that extracted by Johansson et al. [9], suggesting that our extracted β-glucan had higher purity. Therefore, the alkaline solution (1 M KOH) was more suitable for extraction of water-insoluble β-glucan.

The molecular weight of samples is shown in Table 1. The number-average molecular weight (M_n_) of water-insoluble β-glucan was 27,700 g/mol, which was lower than that of water-soluble β-glucan (34,300 g/mol). The weight average molecular weights (M_ws_) were 123,800 g/mol for water-insoluble β-glucan and 119,200 g/mol for water-soluble β-glucan. These results are consistent with the molecular weight of oat β-glucan (65,000–3,100,000 g/mol) reported by Lazaridou et al. [10]. As an important parameter for characterizing the molecular weights of polymers, the M_w_/M_n_ ratios of water-insoluble and -soluble β-glucan were measured. These values were 4.47 and 3.48, respectively, which were higher than that (1.72) reported by Ryu et al. [1], indicating that both water-insoluble and -soluble β-glucan had relatively wide molecular weight distribution.

Previous studies have shown that the molar mass of β-glucan is related to its gelation ability and rheological properties, which affect its physiological functions in human intestine [29]. However, according to the results of Wilson et al. [30], the cholesterol-lowering effects of barley β-glucans with high (1000 kDa) and low (175 kDa) molecular weight did not significantly differ. What is more, animal experiments have shown that molecular weight has no linear relationship with the ability to bind bile acids and fats in vitro [30]. Therefore, the physiological function of insoluble β-glucan cannot be predicted only by molecular weight, its role in human body needs to be further studied.

### 3.2. Oligosaccharide Content (DP3:DP4) of Water-Insoluble β-Glucan

**Figure 2:**
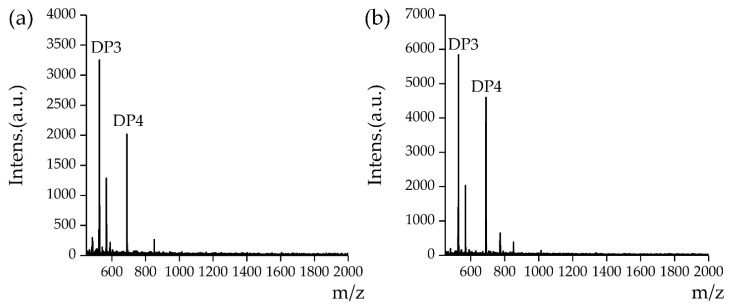
MALDI-TOF mass spectra of β-glucan. (**a**) Water-insoluble β-glucan and (**b**) water-soluble β-glucan. Fragment DP3 (M_w_: 526.91); fragment DP4 (M_w_: 689.16).

The MALDI-TOF spectra of β-glucan (Figure 2) confirmed the presence of DP3 and DP4 as the predominant species. DP3 and DP4 were identified according to literature [24]. The calculated DP3:DP4 molar ratios of water-insoluble and -soluble β-glucan were 2.12 and 1.67, respectively. These results were lower than those reported by Johansson et al. [9] (2.3 and 1.8, respectively) but consistent with the results of Lazaridou et al. [10], who reported values between 1.5 and 2.3 for oat β-glucan. In addition, the results corresponded well with the findings of Izydorczyk et al. [17], who reported that alkali-extracted β-glucan had higher DP3 and DP4 ratio than water-extracted β-glucan.

The DP3:DP4 value of water-insoluble β-glucan was 1.27 times that of water-soluble β-glucan, indicating an increase in cellotriose units. Cellotriose units and other components form helixes through hydrogen bonds, resulting in water-insoluble β-glucan that cannot be extracted by water [31]. Konak et al. [32] found that polysaccharides and other components were easily dissolved under alkaline conditions; therefore, potassium hydroxide reagent was chosen to extract water-insoluble β-glucan.

### 3.3. Spectroscopic Analysis of Water-Insoluble β-Glucan

**Figure 3:**
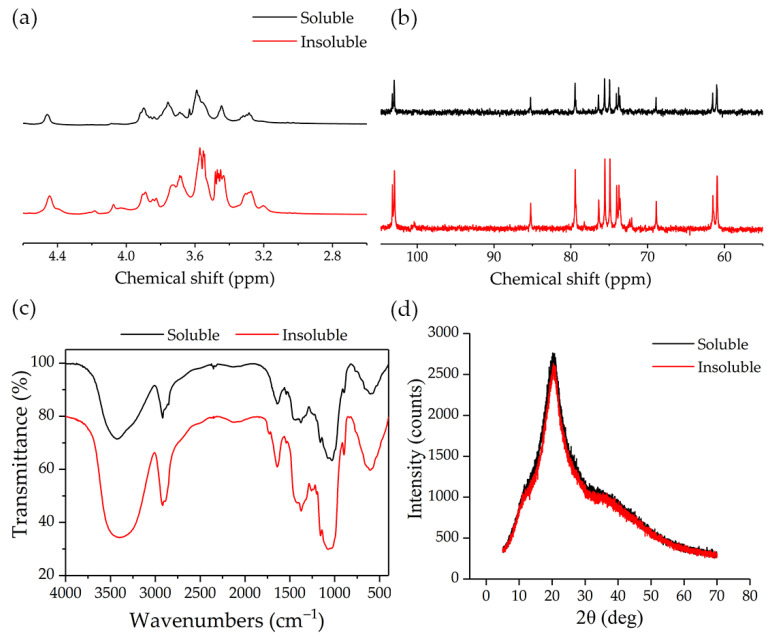
Spectroscopic analysis of water-insoluble β-glucans. (**a**) ^1^H spectrum of β-glucan in D_2_O. (**b**) ^13^C spectrum of β-glucan in D_2_O. (**c**) FT-IR spectra of β-glucan in oat bran. (**d**) X-ray diffraction patterns of β-glucan in oat bran. Soluble, water-soluble β-glucan; Insoluble, water-insoluble β-glucan.

Figure 3 showed the spectra of water-insoluble and -soluble β-glucan. The majority of chemical shifts for the samples were observed within chemical shift δ 2.8–4.7 ppm in ^1^H NMR spectra (Figure 3a). This is a typical polysaccharide signal and consistent with previous reports [33]. Additionally, water-insoluble β-glucan had multiple peaks between chemical shift values of δ 3.6–3.4 ppm, potentially because of the presence of more hydroxyl groups on water-insoluble β-glucan chain, causing overlapping resonances and leading the proton signal to be shifted or lost on the glucose residue.

The ^13^C NMR spectra of both water-insoluble and -soluble β-glucan showed broad resonances in the region 50–110 ppm (Figure 3b). The resonances at 103.22, 74.90, 85.22, 73.72, 76.38, and 61.51 ppm were assigned to C-1, C-2, C-3, C-4, C-5, and C-6 carbons of the β-(1→3) linkage, respectively. The signals at 102.97, 68.88, 74.03, 79.43, 75.57, and 60.96 ppm were assigned to C-1, C-2, C-3, C-4, C-5, and C-6 carbons of the β-(1→4) linkage, respectively [25]. There was little difference from the study of Johansson et al. [21], who reported that the β-(1→3) linkage resonances of C-2, C-3, C-4, and C-5 were 72.5, 87.3, 68.6, and 76.5; this may be due to different reagents used to dissolve samples. No carbon signal was found at 100 ppm, indicating that the absence of starch in β-glucan.

The FT-IR spectra of the two samples are shown in Figure 3c. The spectra for water-insoluble and -soluble β-glucan showed typical signals of glucose polysaccharides. The strong and wide stretching peaks near 3430 cm^−1^ represented -OH stretching, which is the characteristic absorption peak of carbohydrates. The bending vibration of −CH_2_ bond appeared at 2920 cm^−1^. The stretching peaks in the region 1400–1200 cm^−1^ were mainly caused by bending and angular vibration of the saccharide -CH bond. The characteristic stretching peaks in the region from 1200 to 1000 cm^−1^ were probably ascribed to asymmetric stretching vibration peaks of the D-pyranoid sugar ring. The peak at 896 cm^−1^ was the characteristic absorption peak of β-D-glucopyranose [34]. Water-soluble β-glucan spectrum was similar with the results of barley β-glucan studied by Xiang et al. [25]. It was worth noting that there were some differences between water-insoluble β-glucan and -soluble β-glucan in the region 1400–1000 cm^−1^. The interconnection of the bonds in this area was easy to produce strong mutual coupling between various vibrations. We speculated that the spatial position and number of the water-insoluble and -soluble β-glucan bonds may be different, resulting in different stretching peaks.

Figure 3d showed X-ray diffraction of β-glucan samples. The diffraction patterns of water-insoluble and -soluble β-glucan were similar, indicating that they have same crystal structure. The diffraction peak was clearly observed at 2θ = 20.5°, which was different from cellulose (2θ = 22.68°), indicating that β-glucan was not cellulose I type [26,35]. Compared with cellulose, β-glucan showed changes in the physical properties of one-third of its glycosidic bonds. The crystal structure of β-glucan was regenerated cellulose hydrate [31]. In summary, water-insoluble and -soluble β-glucan had similar crystalline structures which were different from that of cellulose.

### 3.4. Microstructure of Water-Insoluble β-Glucan

**Figure 4:**
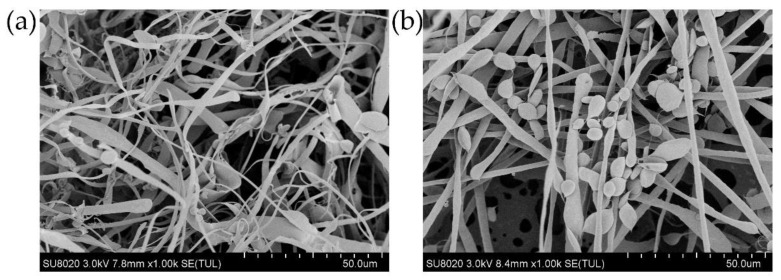
Scanning electron microscopy for (**a**) water-insoluble β-glucan and (**b**) water-soluble β-glucan.

The apparent morphology of β-glucan is affected by drying procedure [36]. In order to minimize damage to the morphology of β-glucan, a vacuum freeze-drying method was used to dry the samples. The surface morphologies of both water-insoluble and -soluble β-glucan included fibrous sheets and small oval-shaped aggregates (Figure 4). Water-insoluble β-glucan contained more curved fibrous sheets, whereas water-soluble β-glucan contained more aggregates. The molecular irregularities of β-glucan were reflected in their water solubility properties. Indeed, the more curved fibrous sheet structures may affect mechanical resistance and water permeability [37], leading to insolubility of water-insoluble β-glucan in water.

### 3.5. Physicochemical Characteristics of Water-Insoluble β-Glucan

#### 3.5.1. Swelling Power, Fat-Binding Capacity, and Gel Texture

The swelling power, fat-binding capacities, and gel texture of water-insoluble and -soluble β-glucan are presented in Table 2. The swelling power of insoluble β-glucan was 7.29 g/g, which was significantly higher than that of water-soluble β-glucan (6.34 g/g). We speculate that due to insoluble β-glucan having higher cellotriose fiber fragments and more curved fiber sheet structure, it may be easier to wrap water through hydrogen bonding in aqueous solution, thus exhibiting better swelling power. Additionally, the fat-binding capacities of water-insoluble and -soluble β-glucan were 3.17 and 3.01 g/g, respectively, similar to reports by Sun et al. [22]. The physiological function of β-glucan was related to its physical and chemical properties. β-glucan swelling in intestine can inhibit the absorption of glucose and fat. Swelling power could predict the cholesterol-lowering ability of fiber, and fat-binding capacity ability may be related to hypolipidemic ability [38,39]. Water-insoluble β-glucan had higher swelling power and fat-binding capacity than water-soluble β-glucan, suggesting that water-insoluble β-glucan may have the potential of lowering cholesterol and blood glucose.

Compared with water-soluble β-glucan, the gel texture values of water-insoluble β-glucan had no significant difference except for Springiness (Table 2). However, we found that -insoluble β-glucan had greater hardness value, indicating that the gel strength of water-insoluble β-glucan was higher. The rigidity of the gel was influenced by both concentration and molar weight [40]. Water-insoluble and -soluble β-glucan had different molecular weights, so this may be the reason why there was difference in the texture. In addition, cereal β-glucan gel was formed by physical cross-linking between molecules [41]. The three-dimensional structure of β-glucan gel was stabilized mainly by multiple inter- and intra-chain hydrogen bonds in the junction zones of the polymeric network [10]. Water-insoluble β-glucan has higher DP3:DP4 value, which indicates there was more cellotriose. Cellotriose fragments could easily form a helical fragment, which was conducive to the stable conformation of the chain conformation. Therefore, water-insoluble β-glucan tended to have greater hardness.

#### 3.5.2. Determination of Rheological Properties

**Figure 5:**
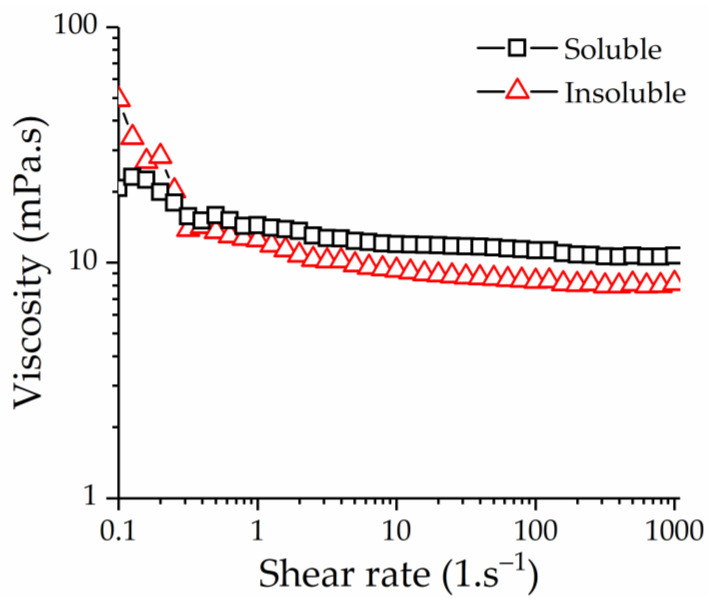
The apparent viscosities of β-glucan at various shear rates with a concentration of 3.0% (*w*/*v*) at 25 °C. Soluble, water-soluble β-glucan; Insoluble, water-insoluble β-glucan.

The viscosity of water-insoluble and -soluble β-glucan was evaluated by rheometry (Figure 5). The viscosities of both water-insoluble and -soluble β-glucan decreased rapidly at low shear rates and then stabilized. These results indicated that β-glucan formed a cohesive and pseudoplastic complex, which is the characteristic of β-glucan. Moreover, the viscosity of water-insoluble β-glucan decreased as the shear rate increased, indicating that the fluidity was increased. Whereas the viscosity of water-soluble β-glucan changed slightly, this result may be related to the presence of additional cellotetraosyl units; these units provide longer segments of 1,4-bond connections, which make the connections between β-glucan tighter and improved resistance against flow and high viscosity [1]. What is more, the influence of β-glucan molecular weight on viscosity was more obvious than that of fingerprint structure [8,42]. The molecular weights and the ratio of DP3:DP4 of water-insoluble and -soluble β-glucan samples were different, resulting in higher viscosity at the beginning of the test. As the shear rate increased, the aggregation state between water-insoluble β-glucan molecules was destroyed, thus exhibiting lower viscosity than water-soluble β-glucan.

### 3.6. Effect of Water-Insoluble β-Glucan on Weight Gain and Lipid Metabolism in HFD-Fed Mice

#### 3.6.1. Effect of Water-Insoluble β-Glucan on Body Weight and Body Fat Content

**Figure 6:**
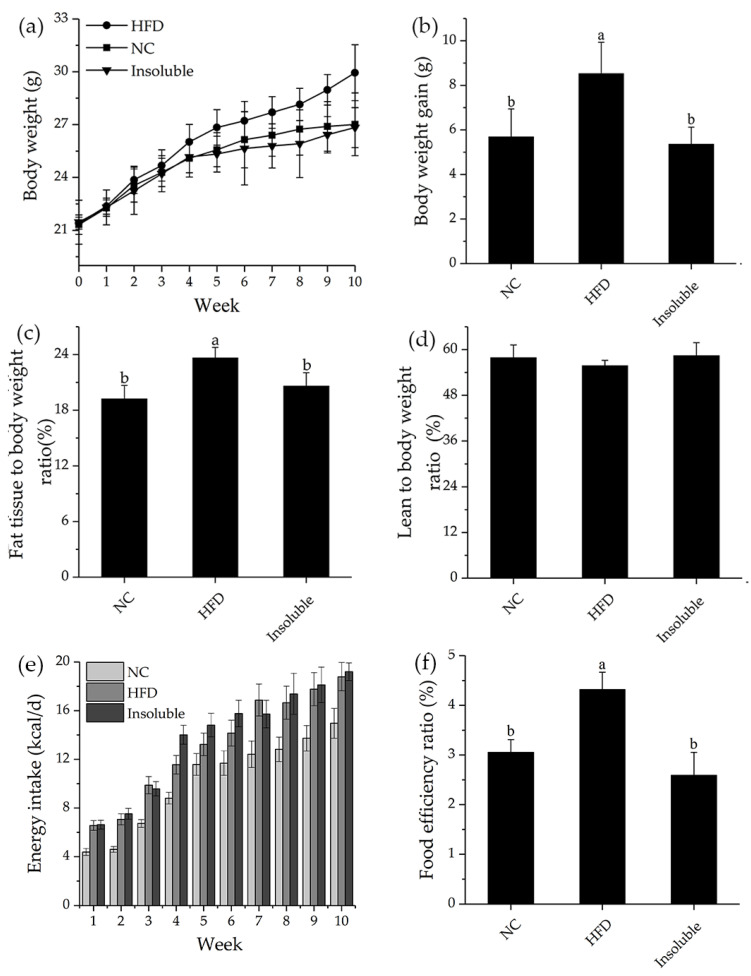
Effect of oral administration of water-insoluble β-glucan on body weight, fat tissue to body weight ratio, lean to body weight ratio, and food intake in HFD-fed mice. (**a**) Changes in body weight over 10 weeks. (**b**) Body weight gain at the end of week 10. Weight gain (%) = [(final weight(g)−initial weight (g))/initial weight (g)] × 100. (**c**) Fat tissue to body weight ratio (%) = fat weight/body weight × 100. (**d**) Lean to body weight ratio (%) = lean meat weight/body weight × 100. (**e**) Daily energy intake of HFD-fed mice. (**f**) Food efficiency ratio (%) = mean body weight gain (g)/mean food consumption (g) × 100. NC, normal control group; HFD, obesity model group; Insoluble, mice fed with HFD and given oral administration of water-insoluble β-glucan. Data are presented as mean ± standard deviation (n = 6). Groups labeled with different letters are different from one another (*p* < 0.05).

The effects of water-insoluble β-glucan on body weight, fat tissue to body weight ratio, and lean meat to body weight ratio of obesity model mice were investigated (Figure 6). There was no significant difference in initial body weight among groups. However, the Insoluble group (oral administration of water-insoluble β-glucan) tended to lower the final body weight of the obese mice after 10 weeks treatment (Figure 6a). What is more, oral administration of water-insoluble β-glucan significantly prevented weight gain compared to HFD group (Figure 6b). In the current study, water-insoluble β-glucan reduced the weight gain of HFD-fed mice and regulated the serum lipid levels of HFD-fed mice, which was consistent with water-soluble β-glucan [43]. The administration of water-insoluble β-glucan in HFD-fed mice resulted in lower relative fat weight compared to the HFD group (*p* < 0.05) (Figure 6c), but it has no effect on lean weight (Figure 6d). There was no significant difference in food intake and food energy intake between HFD and Insoluble groups (Supplementary Materials, Figure S2; Figure 6e), whereas the body weight gain and food efficiency ratio (Figure 6f) were significantly lower in the Insoluble group compared with HFD group (*p* < 0.05). The final body weight was lower in the Insoluble group than in the HFD group. These results indicate that mice in the Insoluble group consumed similar diet and energy as the HFD group, but the Insoluble group had lower food efficiency ratio, which resulted in decreased body weight of HFD-fed mice. Therefore, we speculated that water-insoluble β-glucan may prevent the transport of nutrients to the absorptive surface of intestine by encapsulating them and limiting the mixing of digestive enzymes and their substrates. In conclusion, water-insoluble β-glucan resists obesity in HFD-fed mice by reducing the accumulation of fat in body.

#### 3.6.2. Effect of Water-Insoluble β-Glucan on Serum and Lipid Metabolism in HFD-Fed Mice

**Figure 7:**
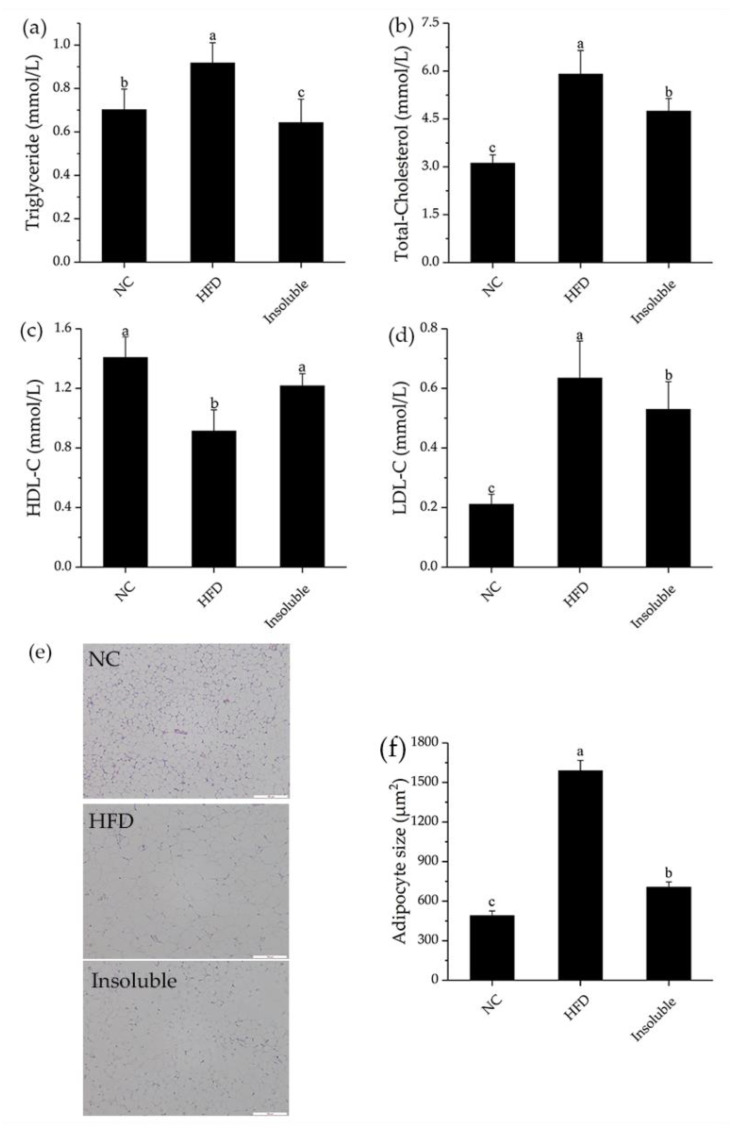
Effect of oral administration of water-insoluble β-glucan on serum biochemical concentrations and shape of epididymis in HFD-fed mice. (**a**) The concentration of triglycerides in serum. (**b**) The concentration of total cholesterol in serum. (**c**) The concentration of HDL-C in serum. HDL-C, high-density lipoprotein cholesterol. (**d**) The concentration of LDL-C in serum. LDL-C, low-density lipoprotein cholesterol. (**e**) Hematoxylin and eosin (H&E) staining of epididymal adipose (200× magnification). (**f**) Adipocyte size = total cell area/the number of cells. NC, normal control group; HFD, obesity model group; Insoluble, mice fed with HFD and given oral administration of water-insoluble β-glucan. Data are presented as mean ± standard deviation (n = 6). Groups labeled with different letters are different from one another (*p* < 0.05).

As shown in Figure 7, water-insoluble β-glucan was effective at significantly improving the serum lipid profile (triglyceride, total cholesterol, high-density lipoprotein cholesterol, and low-density lipoprotein cholesterol levels) (*p* < 0.05). In addition, sections of epididymal adipose tissue of mice in all groups were prepared and observed. From Figure 7e, we could see that the size of adipocytes in HFD group increased significantly compared with NC group, while the adipocytes number decreased. After administration of water-insoluble β-glucan, the number of adipocytes was increased, and the adipocytes size significantly reduced (Figure 7f). The results showed that administration with water-insoluble β-glucan significantly reduced the triglyceride, total cholesterol, LDL content, and adipocytes size, indicating that water-insoluble β-glucan has the potential to improve lipid metabolism and alleviate obesity induced by HFD.

#### 3.6.3. Effect of Water-Insoluble β-Glucan on the Expression of Lipid Metabolism Associated Proteins in Mice

**Figure 8:**
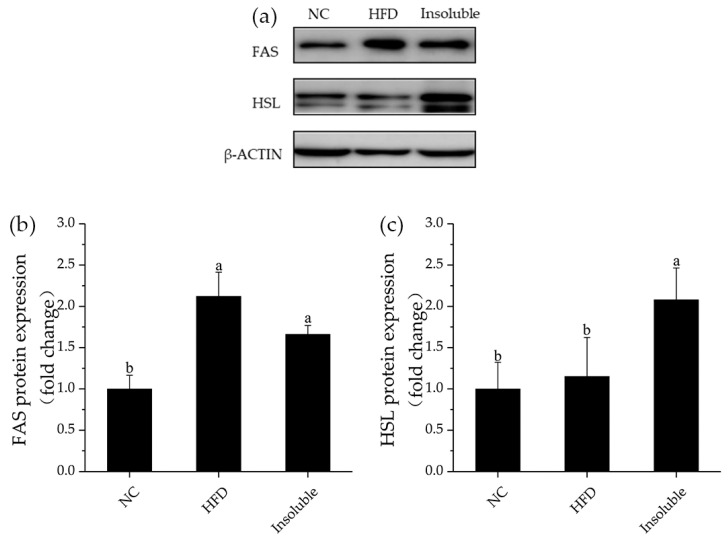
Effect of water-insoluble β-glucan on the expression of lipid metabolism-associated proteins in the liver tissues of HFD-fed mice. (**a**) Representative western blot for FAS and HSL. Quantification of FAS (**b**) and HSL (**c**) protein expression levels. NC, normal control group; HFD, obesity model group; Insoluble, mice fed HFD and given oral administration of water-insoluble β-glucan. Data are presented as mean ± standard deviation (n = 6). Groups labeled with different letters are different from one another (*p* < 0.05).

The lipid metabolism disorder caused by HFD was accompanied by increased lipid synthesis and decreased lipid metabolism, which were manifested by changes in the expressions of related genes such as fatty acid synthase (FAS) and hormone-sensitive lipase (HSL). FAS and HSL, as the key enzymes in lipid metabolism, reflect the synthesis and decomposition of fat in liver. To investigate the mechanism by which water-insoluble β-glucan inhibits lipid accumulation in HFD-fed mice, the protein expression level of FAS and HSL in liver were measured. FAS protein expression levels in Insoluble group were lower than those in HFD group (Figure 8b). In addition, compared with HFD group, HSL protein expression levels in Insoluble group were significantly increased (Figure 8c). The results showed that feeding water-insoluble β-glucan could increase the protein expression of HSL in liver, thus reducing the accumulation of fat. In obese mice, lipid oxidation decomposition was decreased, whereas lipid synthesis was increased. Numerous fatty acids could flow into liver cells, causing lipid accumulation in liver [44]. Our results proved that water-insoluble β-glucan reduced the accumulation and accelerated the decomposition of lipid in liver, thereby having the potential to treat obesity.

## 4. Conclusions

In this study, we showed the structure and function of water-insoluble β-glucan isolated from oat bran. Water-insoluble β-glucan had higher DP3:DP4 ratio compared to water-soluble β-glucan. In terms of surface morphology, water-insoluble β-glucan had more curved fibrous sheet structure than water-soluble β-glucan. Additionally, water-insoluble β-glucan had better swelling power and tended to have better fat-binding capacity than water-soluble β-glucan. Animal experiments have shown that water-insoluble β-glucan could significantly reduce the body weight of HFD-fed mice. The administration of water-insoluble β-glucan significantly improved serum lipids levels and reduced the adipocytes size. Western blotting results showed that feeding water-insoluble β-glucan could increase the protein expression of HSL in liver, thus reducing the accumulation of fat. Taken together, these results proved that water-insoluble β-glucan isolated from oat bran could improve lipid metabolism and accelerate the decomposition of lipid in HFD-fed mice, thereby having the potential to alleviate obesity. Water-insoluble β-glucan may be further developed as a functional food to alleviate obesity in the future.

## Figures and Tables

**Table 1:** Molar masses of water-insoluble β-glucan in oat bran.

Sample	M_n_ × 10^4^ g/mol	M_w_ × 10^4^ g/mol	M_w_/M_n_
Water-insoluble	2.77	12.38	4.47
Water-soluble	3.43	11.92	3.48

**Table 2:** The swelling power, fat-binding capacity, and texture profiles of water-insoluble β-glucan in oat bran.

Sample	Water-Insoluble	Water-Soluble
Swelling power (g/g sample)	7.29 ± 0.01 ^a^	6.34 ± 0.06 ^b^
Fat binding capacity (g oil/g sample)	3.17 ± 0.01 ^a^	3.01 ± 0.05 ^a^
Hardness (N)	3.12 ± 0.04 ^a^	2.08 ± 0.35 ^a^
Adhesiveness (mJ)	2.75 ± 0.16 ^a^	4.62 ± 0.59 ^a^
Cohesiveness	0.22 ± 0.02 ^a^	0.43 ± 0.08 ^a^
Springiness (mm)	2.41 ± 0.24 ^b^	6.10 ± 0.54 ^a^
Gumminess (N)	0.69 ± 0.09 ^a^	0.88 ± 0.01 ^a^

Data are presented as mean ± standard deviation (n = 3). Different letters in the same raw (^a,^ ^b^) indicate significant differences among samples at the *p* < 0.05 level.

## Supplementary Materials

The following are available online at https://www.mdpi.com/article/10.3390/nu13093254/s1, Section S2.2. Separation of Water-Insoluble Dietary Fiber from Oat Bran. Section S2.3. Extraction and Purification of Oat Water-Insoluble β-glucan. Figure S1. Process used for the extraction of (A) insoluble dietary fiber and (B) insoluble β-glucan. Figure S2. Effect of oral administration of water-insoluble β-glucan on daily food intake in HFD-fed mice. Table S1. Formulation of chow diet and high-fat diet (g/kg diet).
