# Supplementary material for: Structural Studies of Water-Insoluble β-Glucan from Oat Bran and Its Effect on Improving Lipid Metabolism in Mice Fed High-Fat Diet
Source: Nutrients. 2021 Sep 18;13(9):3254. doi: 10.3390/nu13093254 (PMC8467107; doi:10.3390/nu13093254)
Supplement: Supplementary file 1 [file nutrients-13-03254-s001.zip › nutrients-1356503-supplementary.pdf]

2.2 *Separation of Water-Insoluble Dietary Fiber from Oat Bran*

Oat bran was pretreated with hot isopropanol and petroleum ether to remove fat. Pre-treated oat bran (100 g) was added with pure water at the ratio of 1:10. The starch in oat bran was removed with amylase and amyloglucosidase. The amylase was pretreated for 20 min at 90 °C to deactivate side reactions. After dispersing the sample in 80℃ water, 1 mL Termamyl SC (~140 KUN/mL) was added and stirred magnetically (RCT basic IKAMAG; IKA, Germany) at 800 r/min for 1 h. Then the pH was adjusted to 4 with acetic acid. One mL α-1,4-Glucan glucohydrolace (300 AGU/mL) was added and stir at 800 r/min and 60℃ for 1 h. Iodine solution test was used to prove that starch has been removed. In order to remove protein from oat bran, we used complex proteinase. The pH of the mixture was adjusted to 3 with acetic acid, then 1 g pepsin (1:3000) was added and stirred at 800 r/min at 40°C for 2 h. Finally, the pH was adjusted to 8. One g trypsin (1:250) was added and stirred at 800 r/min at 40°C for 2 h. The mixture was centrifuged at 3,000 × g for 20 min at 25°C (Thermo Scientific™ Sorvall™ LYNX 6000, Thermo, USA). The precipitate was oat insoluble dietary fiber. The supernatant was precipitated by 70% ethanol which was water-soluble β-glucan concentration. The content of β-Glucan was determined using a Megazyme Mixed-Linkage β-Glucan kit (Wicklow, Ireland). Protein content was analyzed using the Kjeldahl method (N × 5.83).

2.3. *Extraction and Purification of Oat Water-Insoluble β-glucan*

After insoluble dietary fiber was extracted with barium hydroxide, the insoluble material was used for additional extraction with potassium hydroxide (Fraction 1). Finally, the supernatant was separated and precipitated with 70% ethanol to obtain water-insoluble β-glucan mixture (Fraction 2). Then, DEAE-52 cellulose and SephadexG200 were used for purifying water-soluble and -insoluble β-glucan with a Bio-Rad NGC Chromatography system (Bio-Rad Laboratories, Hercules, CA, USA).

For DEAE-52 cellulose experiment, the crude β-glucan was precipitated with 30% ammonium sulfate which was repeated three times. The precipitate was dissolved, and dialyzed against ultrapure water for 72 h (molecular weight cutoff :500 Da). Then the dialysate was centrifuged at 8,000 × g for 5 min, and 5 mL supernatant was loaded into DEAE52 column (10 × 4 cm, Merck Millpore, USA), followed by elution with distilled water. At a flow rate of 0.5 mL/min, 5 mL of eluent per tube was collected. Phenol-sulfuric acid method was used to track the polysaccharide content (the absorbance peak at 490 nm was detected by a microplate reader). The main sugar-containing peak was collected according to the elution curve. For the sample with the most polysaccharide content obtained in DEAE chromatography, two mL of supernatant was injected into SephadexG200 column (100 × 1.6 cm, Merck Millpore, USA), and 0.22 μm filter membrane filtered distilled water was used as the eluent. One column volume was eluted at a flow rate of 0.2 mL/min. Five mL was collected in each tube, and the elution curve was detected by the phenol-sulfuric acid method. The components were collected according to the elution curve.

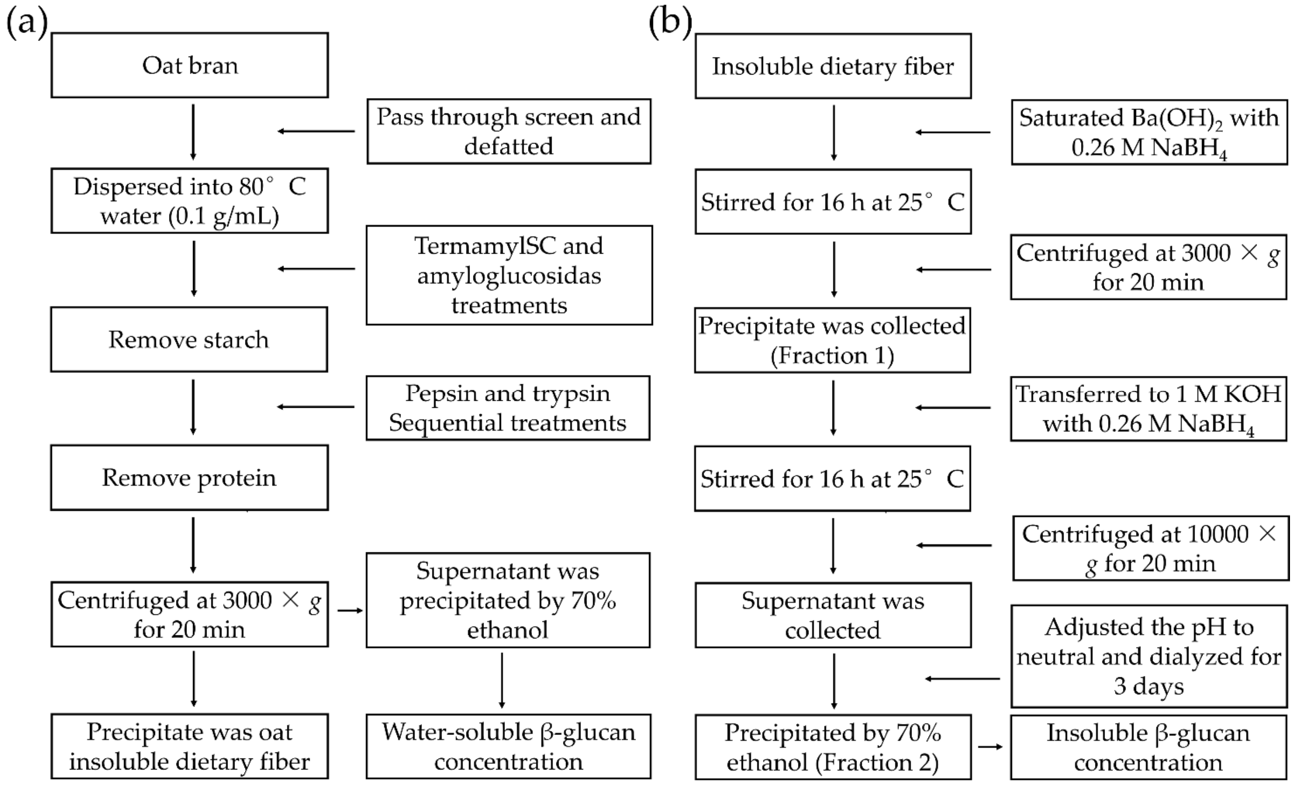

Figure S1. Process used for the extraction of (a) insoluble dietary fiber and (b) insoluble β-glucan.

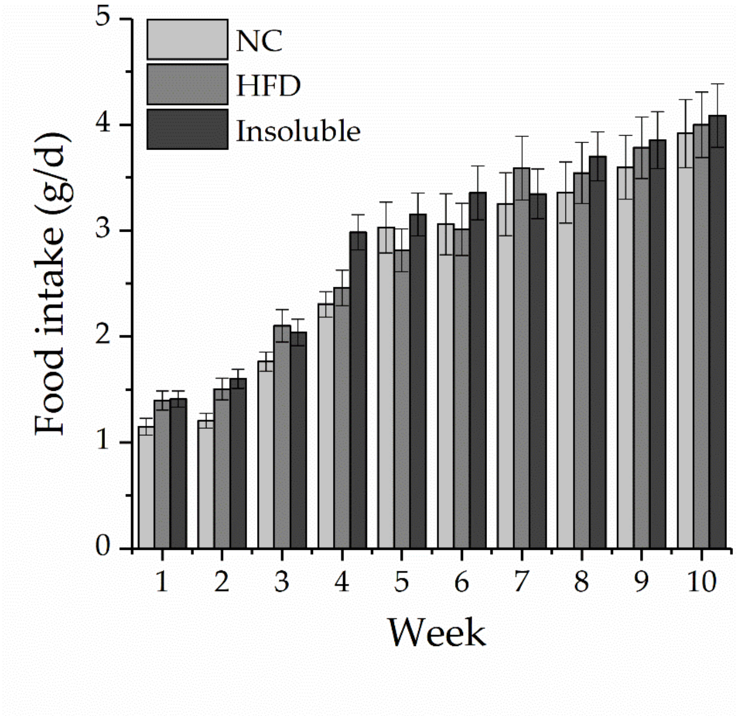

Figure S2. Effect of oral administration of water-insoluble β-glucan on daily food intake of HFD-fed mice.

Table S1. Formulation of chow diet and high-fat diet (g/kg diet).

Class description	Ingredient	Chow diet	High-fat diet
Protein	Casein, Lactic, 30 Mesh	189.56	233.06
	L -Cystine	2.84	3.50
Carbohydrate	Sucrose, Fine Granulated	335.54	206.02
	Starch, Corn	298.56	84.84
	Maltodextrin 10	33.17	116.53
Fiber	Solka Floc, FCC200	47.39	58.26
Fat	Soybean Oil, USP	23.70	29.13
	Lard	18.96	206.84
Mineral	Mineral Mix S10026B	47.39	58.26
Vitamin	Choline Bitartrate	1.89	2.33
	Vitamin Mix V10001C	0.95	1.17
Dye	Dye, Yellow FD&C #5, Alum. Lake 35-42%	0.05	0.06
	Total energy(kcal)	3826.42	4704.21
